# Using Multi-objective Optimization to Identify Dynamical Network Biomarkers as Early-warning Signals of Complex Diseases

**DOI:** 10.1038/srep22023

**Published:** 2016-02-24

**Authors:** Fatemeh Vafaee

**Affiliations:** 1 Charles Perkins Centre, University of Sydney, Sydney, Australia; 2 School of Mathematics and Statistics, University of Sydney, Sydney, Australia

## Abstract

Biomarkers have gained immense scientific interest and clinical value in the practice of medicine. With unprecedented advances in high-throughput technologies, research interest in identifying novel and customized disease biomarkers for early detection, diagnosis, or drug responses is rapidly growing. Biomarkers can be identified in different levels of molecular biomarkers, networks biomarkers and dynamical network biomarkers (DNBs). The latter is a recently developed concept which relies on the idea that a cell is a complex system whose behavior is emerged from interplay of various molecules, and this network of molecules dynamically changes over time. A DNB can serve as an early-warning signal of disease progression, or as a leading network that drives the system into the disease state, and thus unravels mechanisms of disease initiation and progression. It is therefore of great importance to identify DNBs efficiently and reliably. In this work, the problem of DNB identification is defined as a multi-objective optimization problem, and a framework to identify DNBs out of time-course high-throughput data is proposed. Temporal gene expression data of a lung injury with carbonyl chloride inhalation exposure has been used as a case study, and the functional role of the discovered biomarker in the pathogenesis of lung injury has been thoroughly analyzed.

Recent advances in high-throughput technologies have provided a wealth of genomics, transcriptomics, proteomics, and metabolomics data to decipher disease mechanisms in a holistic and also dynamical manner^1^. Such plethora of -omics data has opened new avenues to the research of translational medicine, and particularly facilitated the discovery of novel biomarkers for complex diseases^2^. Biomarkers are indicators of some biological states or conditions, which are often measured and evaluated to examine normal biological processes, pathogenic conditions, disease progression, or pharmacologic responses to therapeutic interventions. The classical approach in biomarker discovery is to identify candidate molecules (e.g., genes, proteins, etc.) whose expression/abundance can classify the disease vs. normal samples, or predict patients’ survival. While molecules are basic components of cellular machinery, a complex disease is not usually the result of the malfunction of individual molecules, but from an interplay of a group of correlated molecules–i.e., a network. Such a viewpoint has raised the concept of network biomarkers which integrates molecular biomarkers with their interactions usually derived from gene expression profiles and/or protein-protein interactions. In recent years, network-based approaches have grown in popularity for their capacity to explain emergent properties such as modularity, phenotypic variation and biological heterogeneity^3^,^4^. However, a cell is a complex dynamical system whose molecular networks continuously change over time. We therefore need an additional level of biomarkers–i.e., dynamical network biomarkers (DNBs)–to portray time-dependent alterations of network biomarkers monitored and evaluated at different stages of disease progression^5^.

One of the recently proposed applications of DNBs is to detect early-warning signals of complex diseases^6^. Evidence suggests that the progression of many complex diseases is abrupt rather than smooth^7^,^8^,^9^. In other words, there is a sudden drastic shift during the process of gradual health deterioration that results in a catastrophic transition to the disease state. So, depending on the disease progression level, the process can be divided into three stages, i.e., normal state, pre-disease state, and disease state^6^: the normal state is a relatively healthy stage–e.g., an incubation period or a chronic inflammation period during which the disease is under control. The pre-disease stage^10^ is defined as the limit of the normal state where the system is at the critical threshold, also known as the “tipping-point”. The pre-disease state is usually reversible to the normal stage under appropriate therapeutic interventions. However, the system becomes hardly reversible if it passes this critical point and enters the disease stage. Hence, detecting the pre-disease state is central for the early diagnosis and consequently for disease prevention.

By using time-course high-throughput -omics data, and based on the principle of bifurcation in dynamical systems, Chen *et al.*^6^ have shown that a DNB can serve as a general early-warning signal of such sudden deterioration, prior to the critical transition to the disease state. They have theoretically shown that when the system reaches the pre-disease state, there exists a group of molecules (i.e., genes or proteins) whose averaged *intra-class* correlation coefficient drastically increases in absolute value, while its averaged *inter-class* correlation coefficient decreases in absolute value. This group also shows a drastic increase in the averaged standard deviation of concentrations of its constituent molecules. If these three properties hold all together, this group is called the dominant group of the system whose emergence is an indicator of the pre-disease state reflecting the transition of the system to the disease state. These criteria once hold, imply that the concentrations of molecules in the dominant group tend to increasingly fluctuate while they behave dynamically in a strongly collective fashion. The dominant group can be portrayed as a subnetwork (i.e., nodes are molecules and the weighted edges are their mutual correlations) which emerges at a particular time-point during the disease progression, and therefore characterizes dynamical features of the underlying system. Hence, it can be considered as a *dynamical network biomarker*.

Detecting DNBs as disease early-warning signals is a recently proposed topic in the field, and the major effort to date has been given to the theoretical derivation of DNB properties and its applications to different diseases. Little effort, however, has been devoted to the computational identification of DNB from high-throughput data. Identifying a DNB is computationally intractable due to the huge number of variables in high-throughput data, and thus, it demands special attention of computer science community to employ effective computational methods to detect DNBs reliably and efficiently.

Here, DNB detection is defined as a multi-objective optimization problem. The overall workflow for DNB identification from high-throughput data is portrayed, and as a case study, DNB and the pre-disease state for the lung injury with carbonyl chloride inhalation exposure were identified using time-course microarray data.

## Methods

### Formal Definition of DNB Criteria

Consider a high-throughput experimental setup where data (e.g., microarray gene expression profiles) is acquired for a set of *K* samples over *T* time-points. Let 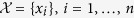 to be the set of *n* molecules under study such that the concentrations of each molecule 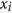 over *K* samples at time-point *t* are represented as


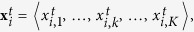


where 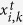 is the concentration of molecule 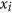 at time *t* for sample *k*. Consequently, a matrix representing concentration vectors of all *n* molecules at time *t* is denoted as


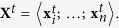


DNB is a group of molecules 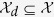 realizing the criteria of the critical transition. 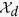 also represents the *leading* network showing these properties. Therefore, a time-point 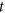 is sought when a group of molecules, 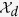, with the following properties is first emerged:

1. 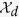 shows a drastic increase in *intra-class* correlation coefficient as defined by Equation 1 where 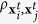 is the Pearson correlation coefficient between two distinct molecules at time *t*.


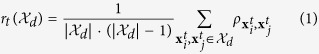


2. It shows a decrease in the *inter-class* correlation coefficient as defined by Equation 2.


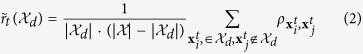


3. Its constituent molecules show drastically increased fluctuation as formalized by Equation 3, where 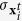 is the standard deviation of molecule 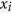 across different samples at time *t*.


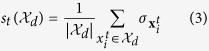


These three properties can be combined together to construct a composite index:


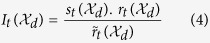


In this formulation, all the mutual correlations are used to derive the composite index for DNB detection, which means that DNB as well as the whole interactome (i.e., the whole set of molecular interactions) are thought to be complete graphs. It implies that co-expressions among any two genes or proteins are *functionally* important regardless of whether the two genes or proteins are directly linked in the actual cellular interactome. However, DNB criteria can be refined for incomplete graphs which may require revisions in the theoretical derivation of DNB properties. The re-derivation of DNB criteria for incomplete graphs is the author’s future plan to extend the current work.

### Multi-objective Optimization

A multi-objective optimization is an optimization problem that involves multiple objective functions, formulated as


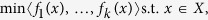


where integer 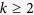 is the number of objectives, 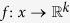 is the vector-valued objective function, and *X* is the solution space. In non-trivial multi-objective optimization problems where the objective functions are *conflicting*, no feasible solution that simultaneously minimizes all objective functions typically exists. Therefore, attention is paid to *Pareto optimal* solutions, i.e., solutions that cannot be improved in any of the objectives without deteriorating at least one of the other objectives. A feasible solution 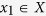 is said to (Pareto) dominate another solution 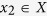, if


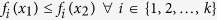


and


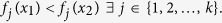


A solution 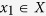 is called Pareto optimal if it is not dominated by any other solution in the solution space^11^. The set of all feasible non-dominated solutions in *X* is referred to as the *Pareto optimal* set, and the corresponding objective vectors are called the *Pareto front*. For many problems, the number of Pareto optimal solutions is enormous and a multi-objective optimizer is usually aimed to identify a representative set of solutions which 1) lie on the Pareto front, and 2) are diverse enough to represent the entire range of the Pareto front^12^.

A popular approach to generate Pareto optimal solutions is to use evolutionary algorithms (EAs). EAs are generic meta-heuristic optimization algorithms whose procedure begins with a population of solutions usually generated at random. It then iteratively updates the current population to generate a new population by the use of four main operators, namely selection, crossover, mutation and elite-preservation. The operation stops when one or more pre-specified termination criteria are met. The use of a population of solutions allows an EA to find multiple optimal solutions, thereby facilitating the solution of multi-objective optimization problems. Furthermore, EAs have essential operators to converge towards a set of non-dominated points which are as close as possible to the Pareto-optimal front, and yet diverse among the objectives^13^.

Currently most evolutionary multi-objective optimization algorithms apply Pareto-based ranking schemes. A standard example is the *Non-dominated Sorting Genetic Algorithm-II* (NSGA-II)^14^. NSGA-II sorts the population into various fronts such that the first front is a completely non-dominant set in the current population (rank 1 individuals), and the second front is only dominated by the individuals in the first front (rank-2 individuals) and this process continues until the entire population is ranked. In addition to the individuals’ ranks, another parameter called *crowding distance* is calculated for each individual. Crowding distance is a measure of how close an individual is to its neighbors. NSGA-II selects individuals based on the rank and the crowding distance.

NSGA-II as well as other evolutionary multi-objective optimizers are *a posteriori* methodologies. In a posteriori methods, a representative set of Pareto optimal solutions is first found and then the decision maker (DM) should choose one of the obtained points using higher-level information. The DM is expected to be an expert in the problem domain although several decision making support methods are developed to aid the DM in the selection of the preferred solutions^15^,^16^,^17^.

### DNB Identification as a Multi-objective Optimization Problem

A DNB shows an *early* yet *strong* signal indicating the emergence of the pre-disease state. Hence, a DNB should meet two objectives simultaneously: 1) it should show a strong signal, measured in terms of the composite index *I*, which is ensued from the sudden deterioration prior to the critical transition, and 2) it is the *leading* network of such critical transition. These two objectives are conflicting in nature as a leading network showing a significantly high signal, is not necessarily the one with the strongest signal throughout the whole spectrum of the disease progression. Hence, DNB identification can be thought of as a bi-objective optimization problem to find a group of molecules whose composite index is maximized in the current time-point while minimized over the prior time-points. In mathematical terms, a multi-objective optimization problem which finds a dominant group of molecules at time *t*, can be formulated as


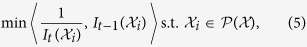


where 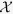 is the set of all molecules and 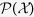 is the power set of 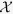 representing the solution space. It is assumed that the disease progression is not therapeutically intervened which implies that a pre-disease stage usually precedes an irreversible disease stage. In other words, the pre-disease stage is not repeated during the disease progression. Therefore, in practice, one should only check time 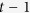 to identify whether a DNB is first emerged at time *t*. If this assumption is voided, Equation 5 should be revised to consider DNB behaviors across all prior time-points (e.g., the second objective can be the average of composite indices for times 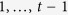.

### DNB Identification: Workflow

**Figure 1:**
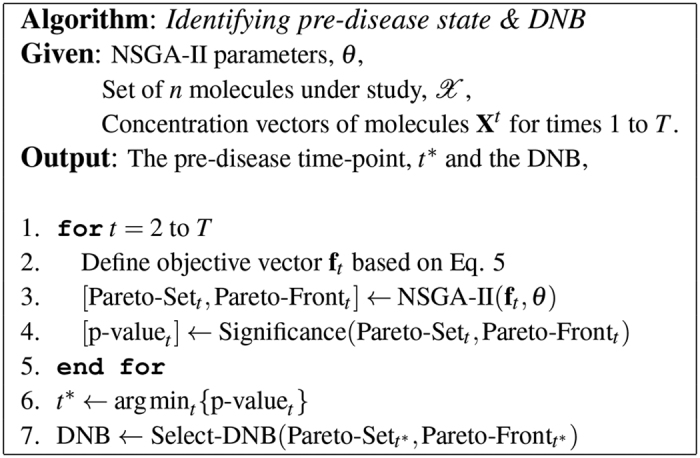
Workflow of identifying the pre-disease state and DNB, using an evolutionary multi-objective optimizer.

Figure 1 shows the workflow of using an evolutionary multi-objective optimization, e.g., NSGA-II, to identify the pre-disease time and the corresponding DNB. The proposed scheme runs NSGA-II for time-points which are feasible candidates of the pre-disease state, usually from the commencement of the experimentation to the time when the system enters the disease state as diagnosed based on samples’ phenotypes or the patients’ symptoms. If no such knowledge is available a priori, *T* is the latest sampling time of the experiment.

Prior to using any evolutionary algorithm, an appropriate representation of individual solutions should be sought. Here, each solution 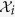 which encodes a group of molecules is represented by a binary string of length 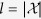 whose bit *i* corresponds to molecule 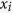. Value 1 indicates that molecule 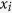 is in the group while 0 indicates that it is not. NSGA-II also requires a number of parameters, *θ*, being set initially such as the crossover and mutation rates, the population size, and the maximum number of iterations.

At each time-point *t*, NSGA-II returns a Pareto set and the corresponding frontiers. The Pareto set then undergoes statistical significance analysis to assess how likely it is to have occurred by sampling error alone. Details of the significance assessment is discussed in the next section.

The pre-disease stage is considered to be the time whose Pareto set *p*-value is the smallest. This generic rule is based on the idea that the lower the *p*-value is, the more significant the Pareto signals are, and thus, the corresponding time-point is more likely to be the pre-disease state. In practice, however, Pareto sets should be more deliberately investigated, particularly when there exists multiple time-points with very close *p*-values. In such cases, some higher-level analyses (e.g., pathway/functional analysis) of Pareto molecules facilitate the discovery of *context-specific* DNBs.

Once the pre-disease state is identified, the final task is to select one of the non-dominated solutions of the corresponding Pareto set as an early-warning biomarker of the disease under study. Here, it is assumed that DM looks for the solution closest to the ideal solution which optimizes all the objectives simultaneously, i.e., both objectives are 0.0:


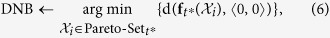


where 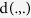 is the point-wise Euclidean distance. Nevertheless, in real problems, the DM who is an expert in the field, should select one of the Pareto solutions using higher-level and context-specific information.

#### Significance assessment of a Pareto set

To estimate the statistical significance of Pareto-Set_*t*_, one first needs to generate a “null” sample which preserves the structure of the observed sample. Accordingly, for each individual in Pareto-Set_*t*_, an *equivalent* random individual was generated which has equal number of molecules (i.e., equal number of 1 bits), but randomly chosen from the pool of molecules under study. Then, for Pareto-Set_*t*_ and its equivalent random set, the distances of the objective vectors of constituent individuals with the optimum 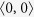 were computed to generate observed and null distributions, respectively. In order to choose an appropriate statistical hypothesis test, the Anderson-Darling goodness-of-fit test for normality^18^ was first run. As none of the observed and random samples followed a normal distribution, a *nonparametric* statistical hypothesis test, i.e., Wilcoxon-Mann-Whitney test^19^ was chosen which returned a *p*-value indicating how likely it is that the two samples come from the same population.

## Results and Discussion

As a case study, the time-course gene expression data of lung injury with carbonyl chloride inhalation exposure (i.e., acute lung injury) was chosen, primarily due to the high temporal resolution of the corresponding dataset. The dataset was obtained from an experiment of a toxic-gas-induced lung injury such as pulmonary edema^20^. Sciuto *et al.* conducted microarray experiments to investigate the molecular mechanism of phosgene-induced lung injury. To acquire control and case samples, two groups of CD-1 male mice were exposed to air and phosgene, respectively. Lung tissue was collected from air or phosgene-exposed mice 0.5, 1, 4, 8, 12, 24, 48, and 72 h after exposure. The details of the experiment are available in the original paper^20^. The dataset was downloaded from NCBI GEO, an open-access repository of high throughput gene expression data^21^. The dataset’s GEO accession number is GSE2565.

### Data Preprocessing

The adopted microarray has 22,690 probe sets originally. The probe sets were mapped to the corresponding NCBI Entrez gene symbols using the GEO annotation file. All probes with no correct corresponding gene symbols were screened out, and multiple probes corresponding to the same gene were aggregated by averaging. 13,214 genes were left for the consequent analysis.

An array usually contains tens of thousands of probes. Many of the corresponding genes, however, do not play any significant role in the context of interest. Therefore, it is important to reduce the dimensionality by filtering out uninformative genes prior to the subsequent learning steps.

Differential expression (DE) analysis (e.g., t-test) is typically used to identify informatory genes whose expression levels significantly change between two sample groups (e.g., case vs. control). Here, at each time-point, a modified version of t-test^22^ was run on the case and control samples, and the *p*-values were obtained. Due to the multiple hypothesis testing, *p*-values were corrected by using the false discovery rate (FDR) estimation^23^. Genes whose *q*-values (i.e., maximal FDR level) were more than 0.01 were filtered out.

Although DE analysis can screen out many genes whose expressions do not significantly change across conditions, it does not assure that the statistically significant differences are also large enough to be biologically meaningful^24^. Hence, fold-change filtering was also performed to choose those differentially expressed genes whose averaged expression in case samples is at least doubled or halved as compared to the controls. This resulted in 304 genes whose expressions significantly and meaningfully changed at least in one time-point.

The last preprocessing step was to normalize case samples with the corresponding control samples in order to make expressions of genes comparable over different time-points. Accordingly, at each time *t*, the expression of each gene 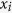 is subtracted from the mean of *x*_*i*_′s expressions across control samples at time *t*, and then divided by the standard deviation of *x*_*i*_′s expressions over the same set of controls.

### Identification of DNB

DNB identification was conducted following the workflow described in Fig. 1, where 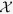 contained 304 genes obtained from the pre-processing step. *T* was considered to be 12 h as disease was well progressed afterwards given that 50–60% mortality was routinely observed after 12 h^20^. NSGA-II parameters were set as follows: population size was set to 500, crossover and (uniform) mutation rates were set to 0.8 and 0.01, respectively. Maximum number of generations was set to 100, and Pareto front population fraction was chosen to be 20%. The workflow was coded in MATLAB R2014b. MATLAB optimization toolbox was used to implement NSGA-II.

**Figure 2:**
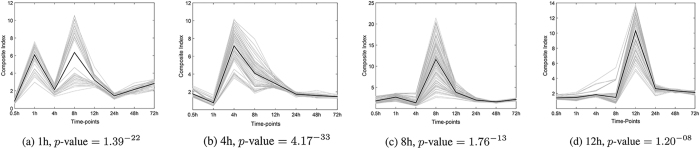
Dynamical behaviors of Pareto solutions (grey lines) identified for different time-points. Black lines represent the averaged trends. The *p*-values of the significance assessment of Pareto sets are provided below the corresponding graphs.

Pareto sets were generated for times 1 h–12 h, and the behaviors of the solutions’ composite indices over the whole spectrum of the disease progression were plotted in Fig. 2. According to the *p*-values, *t* = 4 h is the highest-ranked candidate for the pre-disease state. 4 h is the earliest time-point whose Pareto solutions show strong and yet unique signals. Times 8 h and 12 h show strong signals, but their higher *p*-values indicate less significant shifts from the null distributions. This hypothesizes that the whole system in these time-points is relatively highly-disrupted. The averaged standard deviation of all molecules at time 8 h, 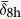, is 3.26, which when compared with 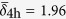, supports the hypothesis of system being holistically inclined in variability. Variability in gene expressions can be generally viewed as having detrimental effects on cellular function with potential implications for disease^25^. Therefore, time-points 8 h and 12 h are potential of being disease states as predicted by DNB identification workflow.

The *p*-value at time 1 h is the second lowest. However, the Pareto solutions at time 1 h have a recurrent pattern with a higher pick at 8 h. This implies that the corresponding molecules are possibly disease genes rather than being the pre-disease biomarkers.

Moreover, historically, the most severe phosgene-induced acute lung injury in this mice model ranges from 4 h to 12 h after exposure^20^ indicating that the system may turn into the disease state after the 4th time period. The prediction here is therefore consistent with the observed disease development.

After identifying 4 h as the pre-disease state, its DNB was chosen to be a Pareto solution whose objective vector was closest to the ideal objectives. The identified DNB contained 16 genes as listed in Table 2.

**Figure 3:**
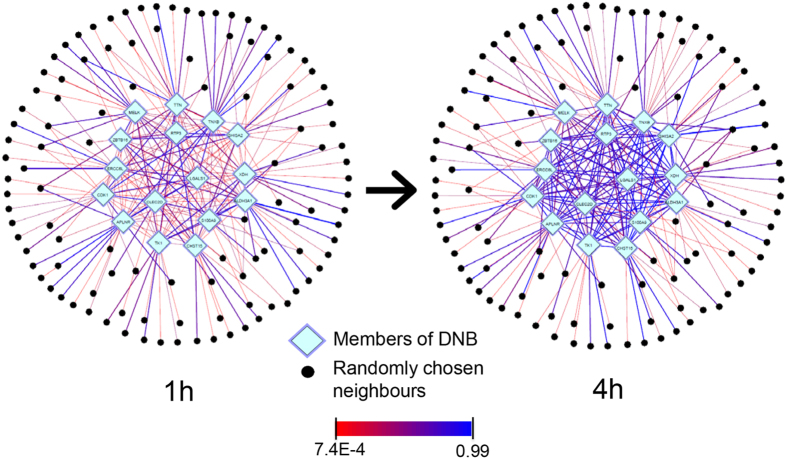
DNB dynamical evolution. Members of DNB are placed in the center surrounded by randomly chosen neighbors; links are colored from red to blue proportional to the correlation values. This Figure clearly shows the emergence of the identified DNB at time 4 h.

In order to visualize how the identified DNB emerged at 4 h, DNB dynamics over the two consecutive time-points of 1 h and 4 h were depicted in Fig. 3. To compare dynamical changes of DNB members vs. other genes, DNB should be shown within the entire co-expression network. However, to avoid too many links obscuring the network visualization, for each DNB molecules, 3% of its neighbors (~10 genes) were randomly chosen. Links are colored from red to blue relative to the expression correlations, where the pure blue (red) corresponds to the highest (lowest) correlation coefficient in the network. Figure 3 clearly shows that DNB forms a strongly correlated observable subnetwork providing a significant signal as the system approaches the critical point.

### Comparison with the Existing Relevant DNB Works

DNBs incorporate temporal alterations with structural information to provide a holistic view of the biological systems and to give a deeper insight into the physiological and pathological mechanisms at the molecular level. DNBs have great significance in disease classification and monitoring, therapeutic response evaluation, early diagnosis and understanding of molecular pathogenesis^5^. As a few examples, a Bayesian network model has been developed to classify subjects with Alzheimer’s disease using longitudinal magnetic resonance data^26^, Yang *et al.*^27^ proposed a dynamic network approach to identify a prognostic signature for acute myeloid leukemia, and a dynamic protein pathway activation of adipose-derived stem cell differentiation identified novel biomarkers of adipocyte differentiation^28^.

The focus of this paper, however, is on one of the key applications of DNBs that is to detect early-warning signals of complex diseases, and to identify the pre-disease state. Dynamical characteristics of the molecular system prior to the critical transition, or the so-called DNB criteria, were theoretically derived by Chen *et al.*^6^ To identify a group of genes best fulfilling DNB criteria, they first clustered genes at each sampling time using a correlation-based agglomerative hierarchical cluster tree. Then, a cluster with the highest change in the composite index between the two consecutive periods was chosen as a DNB, and the corresponding period is regarded as a candidate period of the pre-disease stage. Liu *et al.*^29^ then employed similar methodology to identify DNBs of type 1 diabetes mellitus (T1DM) using temporal gene expression profiles obtained from pancreatic lymph nodes of non-obese diabetic mice samples. Likewise, Li *et al.*^30^ followed a similar approach to identify tissue-specific DNBs associated with type 2 diabetes mellitus (T2DM) progression using gene expression data obtained from multiple tissues of diabetic rat models.

Inspired by DNB core theoretical idea, Yu *et al.*^31^ proposed an *edge network* to exploit higher-order statistics information among molecules. In an edge network, a node is not a molecule but a pair of molecules (i.e. an edge in conventional node networks), and a link represents the relationship between two molecule pairs (i.e. between two edges) rather than between two molecules. By assuming Gaussian distribution on the expression levels of each molecule, an edge network reflects the second-order statistical information of a dynamical system, and thus captures the stochastic dynamics of the original biological system as well as the node network. The computational approach for DNB identification was to first construct an edge network for each sample/subject using fourth-order correlation coefficient estimation. Then, those edges (i.e. molecule pairs) appeared in majority of the subjects’ edge-networks (>75%) were thought to be closely related to the disease development, and their corresponding genes were extracted as DNB genes. The pre-disease time is the time with significant increase in the composite index value of the identified DNB.

Later on, Zeng *et al.*^32^ developed a framework to investigate dynamical organization of molecular modules during early disease development. The proposed workflow was applied on gene expression data of T1DM progression in a mouse model. They identified T1DM pre-disease modules based on DNB criteria as well as modules observed at T1DM advanced stages by cross-tissue gene expression analysis. To identify the DNB and the corresponding pre-disease time, the tissue-specific and time-specific networks are first constructed from the available gene expression data and biological interactions. Then, each context-based network is divided into sub-networks (i.e. modules) using Markov clustering algorithm (MCL)^33^. For each tissue, the pre-disease module was set to be a module with the largest composite index value and the time when the pre-disease module has the largest score was thought to be the pre-disease time.

Recently, Li *et al.*^34^ proposed a model-based framework for DNB identification and analysis of deterioration mechanisms of complex diseases. The workflow began with a network construction by integrating protein interactions with gene expression data, and then using an ODE-based dynamic optimization method to infer the underlying dynamic networks. The network modules were identified using ClusterONE algorithm^35^. “High-influence” modules were then detected based on their average degrees in the whole network. A DNB was identified from high-influence modules when the composite index achieves its largest value.

To investigate the advantage of multi-objective optimization workflow proposed in this work, the identified DNB of acute lung injury (GSE2565 dataset) was compared with those achieved by the existing relevant methodologies as mentioned above. Table 1 lists for each of the compared methods, the predicted DNB size, the corresponding pre-disease time, the intensity of DNB signals at the pre-disease time, and the genes contained in the predicted DNBs.

According to the experimental results, by using the multi-objective optimization approach, the disease initiation signal was detected one step earlier. This is a noteworthy achievement in “predictive medicine” which facilitates earlier therapeutic interventions, and thus improves the chance of disease prevention. Also, according to the biological experiments, the most severe physiological effects occurred within the first 8 hours after exposure, resulting in the increase of the pulmonary edema and decrease of the survival rate (the mortality rate of 50–60% was observed after 12 h). Therefore, the system has potentially entered into the disease stage after 8 hours, and predicting 8 h as the pre-disease state is not well-compatible with the experimental observations. Moreover, the distribution of differentially expressed genes at different time-points (Supplementary Figure S2) demonstrates that the most severe fluctuations in gene expressions commenced at time 4 h verifying the criticality of this time-point. Hence, 4 h cannot be considered a stable healthy state (i.e., a state prior to the pre-disease time) as predicted by other methodologies.

Table 1 also shows sizes of the identified DNBs. Previous studies have shown that very large biomarkers are highly redundant and thus less potential to receive clinical utilities^36^. Also, very small biomarkers do not usually show functional diversity expected in complex diseases. Method 1 identified a DNB containing 220 genes, which is huge as compared to the subsequent methodologies. This method used a hierarchical clustering algorithm with pre-defined number of clusters. Such an approach is prone to produce false-positive biomarkers as all data-points are forced to place in a cluster. Therefore, many of the molecules in the identified DNB might be placed in that group merely because there was no better cluster to fit them in. Method 3, on the other hand, produced a signature containing only three genes all from the same family (heat shock proteins). Although this DNB shows a strong signal at the pre-disease time, it lacks the required functional diversity. Other methods identified DNBs of reasonable sizes.

Lastly, the DNB signal intensity was measured as the escalation of the composite index at the pre-disease time as compared to the previous time-point, i.e., 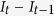, where *t* is the pre-disease time as predicted by the corresponding method. In addition to this, Supplementary Figure S1 shows the temporal behaviors of DNBs predicted by different methods in terms of the change of the corresponding composite indices over time. Theoretically, the composite index of a DNB significantly increases at the pre-disease time as compared to the previous healthy states. The statistical significance of this increase has been assessed using a permutation test as explained in Supplementary Materials. The corresponding *p*-values displayed in Table 1 elucidate the significance of the pre-disease signal of the DNB identified by the proposed optimization approach as compared to those predicted by the former methodologies.

In conclusion, the multi-objective optimization workflow detected a reasonably-sized DNB which predicts the disease initiation earlier in time yet with a strong signal. Together, all these pieces of evidence verify the effectiveness of the proposed methodology as compared to the previous DNB works.

### DNB Functional Role the Pathogenesis of Acute Lung Injury (ALI)

#### The identified DNB triggers the regulation of ALI associated genes

DNB is the leading network of the critical transition which drives the system into the disease state, and thus is strongly linked with casual genes or genes involved in the pathogenesis^37^. A three-step procedure as detailed below was followed to show that the identified DNB could trigger the regulation of genes known to be associated with acute lung injury, and thus corroborates the validity of the DNB’s expected functions.

1. **Extending the Core DNB:** The core DNB (i.e., 16-gene network) was first extended with protein-protein interactions (PPIs). The idea is that DNB proteins may bind to and thus trigger other proteins although mRNAs of interacting partners are not significantly expressed according to the microarray data—possibly due to the fact that the degradation rate of mRNAs is usually much faster than those of proteins^7^. Experimentally validated PPIs were obtained from MppDB^38^ and FpClass training set^39^. FpClass is a data-mining based tool whose training set comprises PPIs detected in at least two experiments. MppDB is a mouse PPI database which provides a referent set of mouse PPIs collected from a variety of online databases. Experimentally validated PPIs were combined with FpClass highly-ranked predicted PPIs (i.e., interactions whose estimated false discovery rates is above 60%) to form a comprehensive PPI database. This database was then queried to retrieve proteins whose interacting partners are in the core DNB, resulting in an extended DNB (EDNB) of 89 molecules as listed in Supplementary Table S1.
2. **Extracting Context-specific Targets of the Extended DNB:** Now the question is that whether the EDNB can drive the regulation of genes relevant to the underlying pathogenesis. The target-genes (TGs) of the EDNB were retrieved from ORTI database, a rich and publicly-available database of experimentally-validated mammalian transcriptional interactions (http://orti.sydney.edu.au/). However, ORTI is a generic database compiling regulatory interactions from different experiments. Therefore, all the retrieved TGs are not necessarily regulated in this particular context/disease. To extract *context-specific* targets, a subset of retrieved TGs which are significantly deregulated (i.e., FDR *q*-value < 0.05) during the disease periods (i.e., 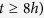 was extracted (293 genes were selected as listed in Supplementary Table S2).
3. **Disease Enrichment Analysis:** The last step was to investigate whether the set of EDNB’s deregulated TGs *enriches* the disease of acute lung injury. In other words, whether the set of EDNB’s deregulated targets statistically *over-represents* genes known to be associated with ALI. First, ALI associated genes were retrieved from DisGeNet database, an expert-curated gene-disease association database^40^. Then, the number of EDNB’s deregulated TGs and ALI associated genes were compared using the right-sided Fisher’s exact test where the p-value for the null hypothesis is computed based the hypergeometric distribution:

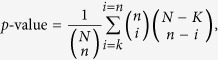


Hypergeometric distribution calculates the statistical significance of having drawn a specific *k* successes (out of *n* total draws) from a population of size *N* that contains exactly *K* successes. Here, *N* is the total number of curated gene-disease associations in DisGeNET (26,522 genes), *n* is the number of EDNB’s deregulated TGs (293 genes), *K* is the total number of genes associated with ALI in DisGeNet (61 genes), and *k* is the number of EDNB’s deregulated TGs associated with ALI in DisGeNet (10 genes). ALI associated TGs were listed in Supplementary Table S3.

#### The extended-DNB enriches ALI associated cellular mechanisms

According to the above-mentioned analysis, DNB genes and their interacting partners (i.e., EDNB) are potential regulators of ALI associated genes, and therefore, are likely to play a role in the pathogenesis of acute lung injury. In order to better understand the underlying cellular mechanisms, EDNB was functionally profiled using Gene Ontology (GO). GO project^41^ provides a system for hierarchically attributing genes or gene products to terms organized in a graph structure (i.e., ontology). The terms are classified into three categories of biological processes, molecular functions and cellular components. In order to determine GO terms statistically overrepresented by EDNB genes, BiNGO tool^42^ was used. BiNGO maps the prevalent functional themes of the queried gene set on the GO hierarchy, and outputs this mapping as a graph in Cytoscape^43^, an open-source software platform for molecular network visualization. BiNGO parameters were set as follows: hypergeometric test was used for the enrichment statistical test and the p-values were adjusted based on Benjamini & Hochberg FDR correction^44^. The significance level was set to 0.01 and the organism was chosen to be Mus musculus.

**Figure 4:**
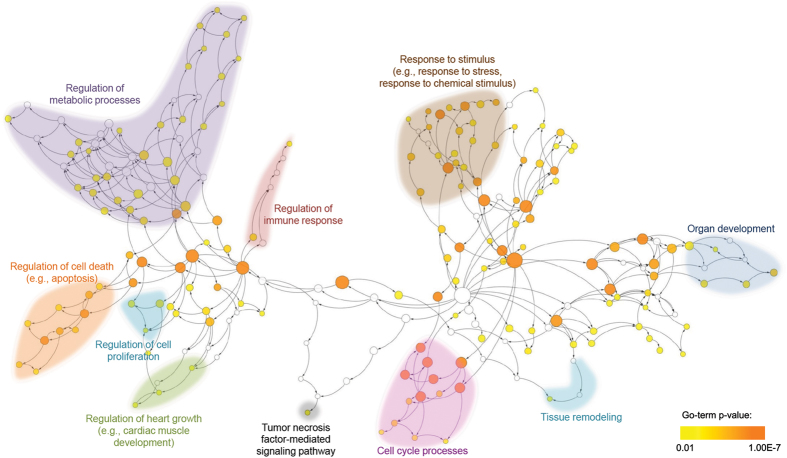
The hierarchy of biological processes enriched by EDNB genes. Nodes are GO terms (i.e., biological processes) and edges shows to the relationships between the terms. Overrepresented biological processes are colored from orange to yellow as the *p*-value increases. White nodes are not significantly overrepresented; they are included to show the colored nodes in the context of the GO hierarchy. The size of a node is proportional to the number of the EDNB genes annotated to the corresponding GO category. Different regions of the enriched ontology were highlighted and functionally annotated based on the underlying biological processes.

Figure 4 shows the hierarchy of biological processes enriched by EDNB genes. Different regions of the enriched ontology were highlighted and functionally annotated based on the underlying biological processes. ALI is the manifestations of a widespread inflammatory response of the lung to direct/indirect insults that may further lead to multiple organ dysfunctions and multiple system failures^45^. Many of the enriched GO terms (e.g., regulation of immune response, response to stimulus, organ development, tissue remodeling) are therefore directly related to ALI initiation and progression processes. In addition, increasing evidence confirms the key role of tumor necrosis factor receptors in the pathogenesis of toxin-induced lung injury^46^, and thus supports the enrichment of tumor necrosis factor-mediated signaling pathway. Evidence also suggests that dysregulation of apoptosis plays a crucial role in the development of ALI and related disorders^47^,^48^, which corroborates the enrichment of GO terms associated with the regulation of cell death and apoptosis. Interestingly, Fig. 4 also demonstrates the significance of regulation of heart growth (e.g., cardiac muscle development). As discussed by Julian^49^, heart muscle reacts to hypoxia by thickening of the muscular wall and hypertrophy (abnormal enlargement) of cardiac myocytes. Hence, the heart response to ALI-induced hypoxia can be the pathological reason for the enrichment of the heart growth regulation.

Overall, the enriched GO terms fairly support cellular processes underlying ALI. Further investigations of genes annotated with these GO terms (as provided by Supplementary Table S4) elucidate early responders/mediators of ALI pathogenesis.

#### Literature supports functional roles of DNB genes in the ALI pathogenesis

The GO term enrichment analysis verifies the functional relevance of DNB genes and their interacting partners as a *set*. In order to further support the biological implications of the *individual* DNB genes, the scientific literature has been manually searched for corroborating information. Table 2 lists 16 genes of the predicted DNB. The existing literature supports the functional role of many of these genes or their family members in the pathogenesis of ALI or relevant disorders. Some of the existing evidence is listed below which elucidates the biological implications of the individual DNB genes and directs subsequent experimental verification:

- **Aldehyde dehydrogenases** are a group of enzymes participating in a wide variety of biological processes including the detoxification of exogenously and endogenously generated aldehydes^50^. Class-2 aldehyde dehydrogenase (mitochondrial) is known to play a role in hyperoxic lung injury by reducing cell death in lung epithelial cells^51^.
- **Apelin** is a group of small peptides which are binding to a G-protein-coupled APJ receptor expressed at the surface of some cells. The apelin and APJ receptor are upregulated during tissue injury^52^. A recent report showed that apelin-APJ signaling pathway is an endogenous anti-injury and organ-protective mechanism activated during acute lung injury^53^.
- **Cyclin-dependent kinase 1** (Cdk1) is a highly conserved protein that functions as a key player in cell cycle regulation. Cdk1 inhibition was shown to reduce lung damage in a mouse model of ventilator-induced lung injury^54^.
- **Carbohydrate sulfotransferases** (Chst family) are sulfotransferase enzymes that are functioning in a wide range of cellular processes, from structural purposes to extracellular communications. The role of small interfering RNA (siRNA) targeting Chst3 has been studied in mouse model with pulmonary emphysema (i.e., lung injury caused by cigarette smoke). It has been shown that Chst3 siRNA diminishes accumulation of excessive macrophages and the mediators, and thus accelerates the functional recovery from the injury^55^.
- **C-type lectin** (Clec) is a type of carbohydrate-binding protein domain known as lectin. Proteins with C-type lectin domains have functional roles in immune response to pathogens and apoptosis^56^. A recent study employed an expression-based genome-wide association approach and identified C-type lectin domain family 4, member D (Clec4d) gene and C-type lectin domain family 4, member E (Clec4e) gene as new biomarkers of acute lung injury^57^.
- **Tenascins** are extracellular matrix glycoproteins. Tenascins have anti-adhesive effects. They are abundant in the extracellular matrix of developing vertebrate embryos and reappear around healing wounds. Tenascin-C is the most intensely studied member of the family and has been shown to be an important mediator of TGF-*β*-mediated fibrosis in the pathogenesis of acute lung injury^58^.
- **Xanthine dehydrogenase** (Xdh) is a protein involved in the oxidative metabolism of purines. A number of studies have supported the role of XDH in the pathogenesis of acute lung injury, hypoxia and relevant diseases^59^,^60^.

In summary, the statistical analyses of the functional role of the identified DNB together with the manual literature search for relevant information strongly support the effectiveness of the proposed methodology from the biological perspective.

## Additional Information

**How to cite this article**: Vafaee, F. Using Multi-objective Optimization to Identify Dynamical Network Biomarkers as Early-warning Signals of Complex Diseases. *Sci. Rep.* **6**, 22023; doi: 10.1038/srep22023 (2016).

## Supplementary Material

Supplementary Information

## Figures and Tables

**Table 1:** Comparison of DNBs of acute lung injury (ALI) and the corresponding pre-disease times predicted by different methodologies (GSE2565 dataset).

Method	*t*_*pre−disease*_	DNB size	*I*_*t*_ *− I*_*t−*1_ (*p*-value)	DNB genes
Method 1 (Chen *et al.*)^6^	8 h	220	3.1 (0.015)	See Supplementary file 1 of the original paper
Method 2 (Yu *et al.*)^31^	8 h	27	2.89 (0.115)	Anln, Asns, Atf3, Bag3, Ccnb1, Cdc20, Cdk1, Cenpa, Cep55, Cks1b, Dnaja1, Dnaja4, Dnajb1, Dnajb4, Gsta2, Hspa1b, Hspd1, Maff, Pbk, Prc1, Spag5, Spp1, Tuba4a, Txnrd1, Ube2c, Uhrf1, Birc5
Method 3 (Zeng *et al.*)^32^	8 h	3	35.82 (0.002)	Hspa8, Hspb1, Hsph1
Method 4 (Li *et al.*)^34^	8 h	25	0.24 (0.909)	Aldoa, Arhgef12, Bnip3, Esd, Gtf2f2, Hk2, Ldha, Papolg, Pard6b, Pcf11, Pgd, pgk1, Pkm2, Pkp3, Ppl, Prkci, Ptbp1, Rhoj, Rhou, Scel, Taldo1, Thoc4, Tkt, Tpi1, U2af1
Current Method	**4 h**	16	5.063 **(0.0)**	See Table 2

Information for Method 1 and Method 4 were extracted from the original papers. Method 3 has provided the java source code of the proposed algorithm. The code was run on ALI data, and the identified modules were extracted for consequent analyses. Method 2 was reproduced for ALI database. Methods are chronologically sorted.

**Table 2:** List of 16 genes included in the identified DNB.

Gene symbol	Gene description	Gene symbol	Gene description
Aldh3a1	aldehyde dehydrogenase 3 family member A1	Rtp3	receptor transporter protein 3
Aplnr	apelin receptor	S100A9	S100 calcium binding protein A9
Cdk1	cyclin-dependent kinase 1	Shisa2	shisa homolog 2 (Xenopus laevis)
Chst15	carbohydrate (N-acetylgalactosamine 4-sulfate 6-O) sulfotransferase 15	Tk1	thymidine kinase 1
Clec2d	C-type lectin domain family 2, member d	Tnxb	tenascin XB
Ercc6l	excision repair cross-complementing rodent repair deficiency complementation group 6 - like	Ttn	titin
Lgals1	lectin, galactose binding, soluble 1	Xdh	xanthine dehydrogenase
Melk	maternal embryonic leucine zipper kinase	Zbtb16	zinc finger & BTB domain containing 16
