# Supplementary material for: Using Multi-objective Optimization to Identify Dynamical Network Biomarkers as Early-warning Signals of Complex Diseases
Source: Sci Rep. 2016 Feb 24;6:22023. doi: 10.1038/srep22023 (PMC4764930; doi:10.1038/srep22023)
Supplement: Supplementary Information [file srep22023-s1.pdf]

Supplementary Materials

Using Multi-objective Optimization to Identify Dynamical Network Biomarkers as Early-warning Signals of Complex Diseases

Fatemeh Vafaee [1, 2]

[1]Charles Perkins Centre, University of Sydney, Sydney, Australia
[2]School of Mathematics and Statistics, University of Sydney, Sydney, Australia
fatemeh.vafaee@sydney.edu.au

Table of Contents

Supplementary Texts 2
Permutation test to assess the significance of a DNB's signal intensity 2
Supplementary Figures 3
Figure S1 3
Figure S2 4
Supplementary Tables 5
Table S1 5
Table S2 6
Table S3 7
Table S4 8

Supplementary Texts

Permutation test to assess the significance of a DNB's signal intensity

In order to assess the significance of the elevation of a DNB composite index at the pre-disease time, a permutation test has been conducted for each of the compared methods. The test statistic is $\triangle I = I_t - I_{t-1}$, where t is the pre-disease time. For a DNB of size n predicted by a compared algorithm, the distribution of the test statistic under the null hypnosis is obtained by randomly generating a group of n genes from the gene pool (i.e., the set of all differentially expressed genes), and repeating this process for 1000 times. The test statistic is then computed for each of the "random DNBs" using the pre-disease time as predicted by the compared algorithm. The p-value is estimated as the fraction of random DNBs whose test statistic is equal or higher than the $\triangle I$ of the DNB of the compared algorithm.

Supplementary Figures

Figure S1

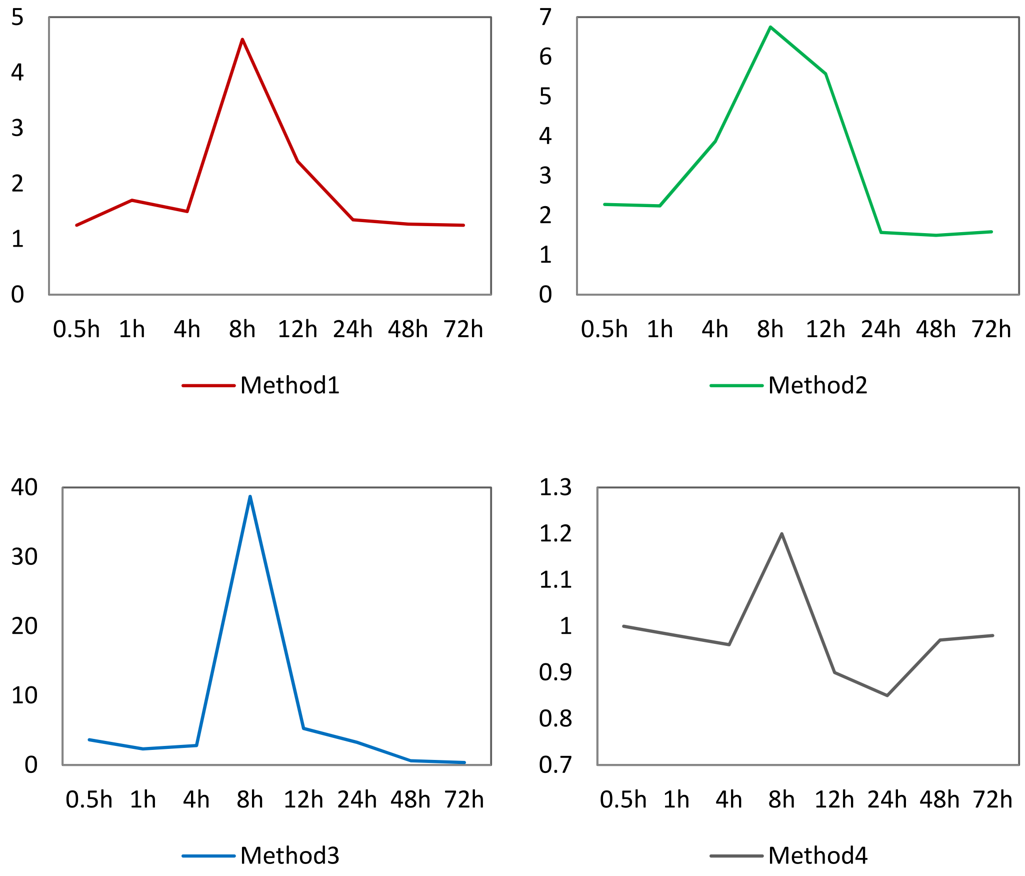

Figure S1: Temporal behaviors of DNBs predicted by different methods in terms of the change of the corresponding composite indices over time; Method 1: [Chen *et al*, 2012], Method 2: [Yu *et al*, 2013], Method 3: [Zeng *et al*, 2014], Method 4: [Li *et al*, 2015]. Information for Method 1 and Method 4 were extracted from the original papers. Method 3 has provided the java source code of the proposed algorithm. The code was run on acute lung injury (ALI) data, and the identified modules were extracted for consequent analysis. Method 2 was reproduced for ALI database.

Figure S2

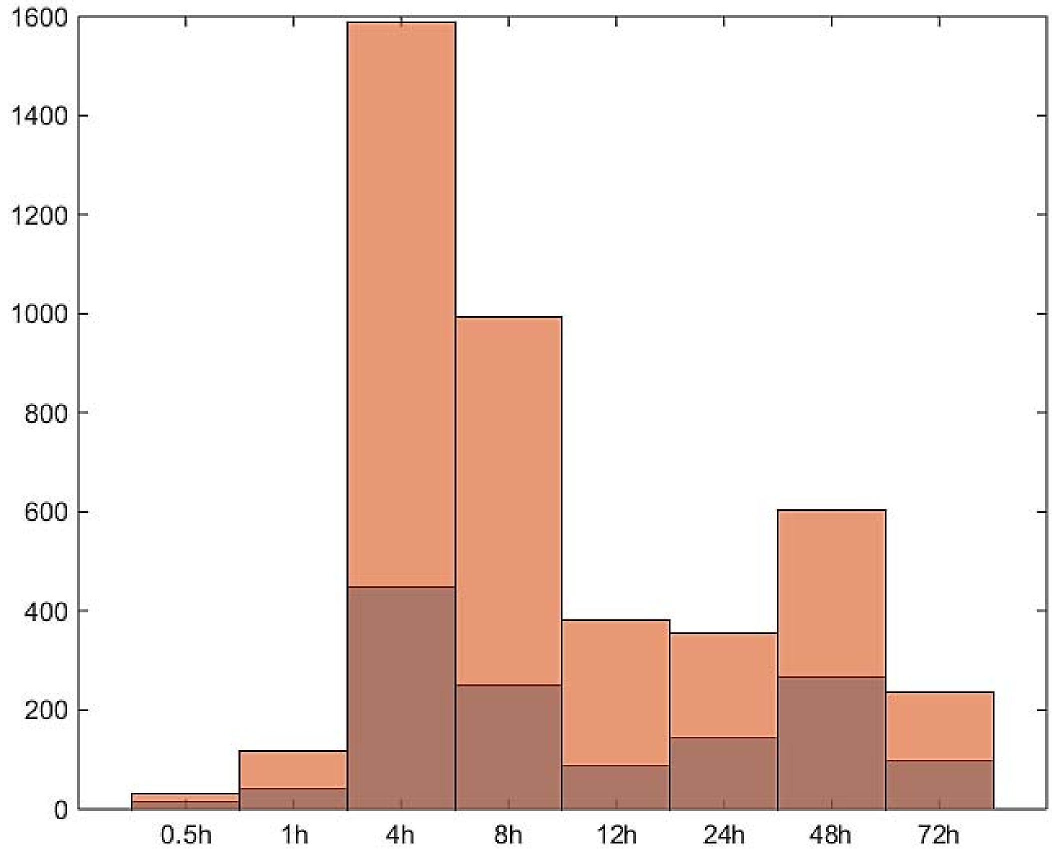

Figure S2: Distribution of genes deregulated at different time-points. Each bar of the histogram shows the number of genes whose deregulation commenced at the corresponding time. The significance is assessed according to the FDR q-value and the threshold is set to **0.05** in pale orange histogram and to **0.01** in dark orange histogram.

Supplementary Tables

Table S1

Table S1: List of 89 genes included in the extended DNB (EDNB).

Genes in Extended DNB			
ALDH3A1	ECT2	LGALS1	SHISA2
ANLN	EGR1	LGALS3	SLC4A1
APLNR	ERCC6L	LTF	SNCA
ASF1B	FCER1G	MAD2L1	SOCS3
AURKA	FKBP5	MCM5	SPAG5
BID	FOS	MELK	SPC25
BIRC5	GADD45G	MKI67	STEAP3
CCNB1	HELLS	MYC	STIL
CCNB2	HMGB2	NCAPG2	TACC3
CCNF	HSP90AA1	NUF2	TGFBI
CDC20	HSPA1B	PBK	TK1
CDCA5	HSPA8	PLK1	TK1
CDK1	HSPA9	PMAIP1	TNXB
CDKN2C	HSPB1	PRC1	TOP2A
CENPA	HSPD1	RACGAP1	TOP2A
CEP55	HSPH1	RAD51	TPX2
CHST15	JUN	RRM2	TRIP13
CKS1B	KIF20A	RTP3	TTN
CKS2	KIF23	S100A4	TUBA4A
CLEC2D	KRT18	S100A8	UBE2C
COL6A3	KRT8	S100A9	UHRF1
CTSK	LCN2	SHCBP1	XDH, and ZBTB16

Table S2

Table S2: List of 293 target genes (TGs) of the extended DNB significantly deregulated after time 4h.

TGs de-regulated in t>4h (FDR p-value <0.05)						
ABCA1	CHRNB1	FUT1	LZTR1	NID1	PSMD3	SRF
ABCB10	CLN3	FXR1	MAFF	NME1	PSMD5	SRI
ABCB6	CLU	GADD45A	MAGOH	NPM1	PTGER2	SRM
ACE	CNPY2	GALC	MAN2A1	NQO1	PTGES3	SRPR
ACTR3	CNTN1	GAPDH	MAPRE2	NR1D1	PTGS2	STOM
ACYP2	COL1A1	GCLC	MBD1	NR3C1	PTOV1	SYN2
ADCY9	COL5A1	GCLM	MCCC2	NRF1	PTP4A1	TBXA2R
ADRB1	COPS2	GDF15	MCL1	NTHL1	PTPN6	TFF1
ALDH9A1	COPS3	GJA1	MCM3	ODC1	PTPRF	TGFB1
ALDOA	COX7A2L	GSS	MDM2	PA2G4	RARA	TGFB2
AMD1	CR2	GSTO1	ME1	PCF11	RARB	TGFB3
AMPD3	CRADD	HBEGF	MEIS1	PCMT1	RBM3	TGM1
APP	CSTB	HERPUD1	MEN1	PCNA	RBM8A	THBS1
AREG	CXCR4	HEY1	MFNG	PDE6D	RFC2	TIMP1
ARF4	CYR61	HIF1A	MGEA5	PDGFA	RFX5	TIMP2
ASCL2	DBI	HMOX1	MGST1	PDGFB	RGS2	TNC
ATF3	DBP	HNRNPA1	MKNK1	PDGFRB	RNF4	TOMM20
ATF4	DDIT3	HNRPDL	MMP14	PDK2	RPL10	TOP1
ATP1A2	DDX1	HOXA5	MMP15	PECAM1	RPL15	TPR
ATP2A2	DGCR6	HOXB2	MMP19	PKM2	RPS6KA2	TRIM25
BAZ1B	DLEU2	HSD11B2	MMP2	PLA2G4A	SARS	TSC2
BCL2	DPYSL3	HSPA1A	MMP3	PLAT	SARS2	TSHB
BCL3	E2F1	HSPA8	MMP8	PLAUR	SCNN1B	TYK2
BID	EAPP	HSPD1	MSN	PLCB3	SEC23B	U2AF1
BMP4	EDN1	HSPE1	MSR1	PMAIP1	SERPINE1	UBA52
BRD7	EGR1	IER2	MTCH1	PMVK	SERPINE2	UBE2D3
CAPZA1	EIF2S1	IFNAR1	MUC1	POLA2	SFTPA1	UBE2G2
CBS	EIF4A1	IFRD2	MUC5B	POLE3	SFTPD	UCHL1
CBX5	EIF4E	ING1	MYB	PON1	SIRT1	USO1
CCL2	ESD	ISG20	MYC	POR	SLC20A1	USP11
CCRL2	ETF1	ITGA2	MYCL1	PPARG	SLC31A1	VAPA
CCT5	ETS1	ITGA5	MYCT1	PPP2CA	SLC39A8	VASP
CCT7	ETS2	JUN	NAB2	PRDX3	SLC3A2	VCAM1
CCT8	F3	JUNB	NAP1L1	PRDX6	SLC9A3R2	VCP
CD14	FABP4	KLK1B4	NCBP2	PREP	SMAD4	VEGFA
CD82	FANCA	LAIR1	NCL	PRKCSH	SNRPB	VHL
CD9	FASN	LAMA4	NDRG1	PRKDC	SOD2	VIM
CDKN1A	FGFR4	LAMP1	NDUFB5	PSAP	SPAST	WDR36
CDKN2B	FLT1	LAMP2	NFAT5	PSMA1	SPIB	XDH
CEBPB	FOS	LDLR	NFE2L1	PSMA5	SPINT1	ZFP36
CEBPD	FOSL1	LHB	NFIL3	PSMB5	SPP1	ZFP36L2
CEBPZ	FUS	LMNA	NFKBIL1	PSMD14	SQSTM1	

Table S3

Table S3: List of 61 genes in DisGeNet related to acute lung injury. Those genes which are among the deregulated TGs of the extended DNB are colored in red.

Genes related to acute lung injury in DisGeNet		
ACVR1	GSTM1	NOS2
ADRA2A	GSTP1	P2RY12
AGER	GSTT1	PECAM1
ALB	ICAM1	PLA2G2A
APOA1	IFNG	PLAU
ASCL1	IL10	PPARG
CAT	IL13	PTGS2
CCL2	IL18	SCGB1A1
CCL4L1	IL1B	SERPINE1
CXCL3	IL1RL1	SOD3
CXCL6	IL1RN	TFF1
CYP1A1	IL2	TFF2
EDN1	IL6	TGFB1
EPHX2	ITGB2	TIRAP
F2	KDR	TLR4
FAS	MMP7	TNF
FASLG	MST1R	TXN
FCGR1A	MTHFR	VEGFA
FCGR2B	MYLK	VIP
GJA1	NFE2L2	
GSR	NOD2	

Table S4

Table S4: GO terms (corrected p-value < 0.01) enriched by the extended DNB using BiNGO tool.

GO-ID	corrected p-value	Description	Genes in test set
**51301**	8.68E-29	cell division	CKS1B, PRC1, AURKA, ANLN, CEP55, ZBTB16, SPC25, NCAPG2, TOP2A, CDCA5, HELLS, ERCC6L, CDK1, CCNF, TPX2, NUF2, BIRC5, CDC20, UBE2C, RACGAP1, MCM5, CCNB1, CCNB2, MAD2L1, PLK1, SPAG5
**7049**	7.83E-28	cell cycle	STEAP3, CKS1B, PRC1, AURKA, ANLN, CEP55, SPC25, CDKN2C, NCAPG2, CDCA5, HELLS, ERCC6L, TRIP13, CDK1, MKI67, CCNF, TPX2, NUF2, CDC20, BIRC5, RACGAP1, UBE2C, TACC3, RAD51, CCNB1, UHRF1, CCNB2, MAD2L1, PLK1, SPAG5, CKS2
**279**	8.16E-28	M phase	CDK1, MKI67, CCNF, NUF2, TPX2, CDC20, BIRC5, AURKA, ANLN, CEP55, TACC3, UBE2C, RAD51, CCNB1, SPC25, CCNB2, MAD2L1, PLK1, NCAPG2, SPAG5, CKS2, CDCA5, HELLS, TRIP13, ERCC6L
**22403**	1.32E-26	cell cycle phase	CDK1, MKI67, CCNF, NUF2, TPX2, CDC20, BIRC5, AURKA, ANLN, CEP55, TACC3, UBE2C, RAD51, CCNB1, SPC25, CCNB2, MAD2L1, PLK1, NCAPG2, SPAG5, CKS2, CDCA5, HELLS, TRIP13, ERCC6L
**9987**	3.93E-26	cellular process	STEAP3, PRC1, SNCA, S100A9, AURKA, PMAIP1, TTN, HSPH1, FOS, CDKN2C, TGFBI, LTF, SLC4A1, TOP2A, MYC, CDCA5, EGR1, CDK1, HSP90AA1, SOCS3, CCNF, TPX2, PBK, TACC3, UBE2C, ECT2, MCM5, RAD51, UHRF1, KRT18, MAD2L1, SPAG5, JUN, RRM2, GADD45G, TUBA4A, MELK, BID, XDH, CKS1B, HMGB2, FKBP5, ANLN, HSPA1B, CEP55, ZBTB16, ALDH3A1, TK1, SPC25, CENPA, NCAPG2, KRT8, FCER1G, ASF1B, HELLS, HSPA8, TRIP13, ERCC6L, HSPA9, TNXB, MKI67, LGALS3, LGALS1, NUF2, CDC20, BIRC5, RACGAP1, CCNB1, LCN2, CCNB2, PLK1, CKS2, HSPD1, KIF20A
**6996**	2.43E-25	organelle organization	BID, HMGB2, S100A9, SNCA, AURKA, ANLN, PMAIP1, HSPA1B, CEP55, TTN, SPC25, NCAPG2, CENPA, KRT8, ASF1B, TOP2A, MYC, CDCA5, HELLS, ERCC6L, CDK1, CCNF, NUF2, TPX2, CDC20, BIRC5, TACC3, UBE2C, CCNB1, CCNB2, MAD2L1, PLK1, SPAG5, JUN
**22402**	4.75E-25	cell cycle process	CDK1, MKI67, CCNF, NUF2, TPX2, CDC20, BIRC5, AURKA, ANLN, CEP55, TACC3, UBE2C, RAD51, CCNB1, SPC25, CCNB2, MAD2L1, PLK1, NCAPG2, SPAG5, CKS2, CDCA5, HELLS, TRIP13, ERCC6L
**16043**	6.19E-25	cellular component organization	BID, HMGB2, S100A9, SNCA, AURKA, ANLN, CEP55, PMAIP1, HSPA1B, TTN, SPC25, CENPA, NCAPG2, KRT8, TGFBI, FCER1G, ASF1B, TOP2A, MYC, CDCA5, HELLS, ERCC6L, CDK1, TNXB, LGALS3, CCNF, NUF2, TPX2, CDC20, BIRC5, TACC3, UBE2C, ECT2, CCNB1, LCN2, CCNB2, MAD2L1, PLK1, SPAG5, JUN, RRM2, TUBA4A
**280**	6.95E-24	nuclear division	CDK1, CCNF, NUF2, TPX2, CDC20, BIRC5, AURKA, ANLN, CEP55, UBE2C, CCNB1, SPC25, CCNB2, MAD2L1, PLK1, SPAG5, NCAPG2, CDCA5, HELLS, ERCC6L
**7067**	6.95E-24	mitosis	CDK1, CCNF, NUF2, TPX2, CDC20, BIRC5, AURKA, ANLN, CEP55, UBE2C, CCNB1, SPC25, CCNB2, MAD2L1, PLK1, SPAG5, NCAPG2, CDCA5, HELLS, ERCC6L
**87**	7.03E-24	M phase of mitotic cell cycle	CDK1, CCNF, NUF2, TPX2, CDC20, BIRC5, AURKA, ANLN, CEP55, UBE2C, CCNB1, SPC25, CCNB2, MAD2L1, PLK1, SPAG5, NCAPG2, CDCA5, HELLS, ERCC6L
**48285**	8.86E-24	organelle fission	CDK1, CCNF, NUF2, TPX2, CDC20, BIRC5, AURKA, ANLN, CEP55, UBE2C, CCNB1, SPC25, CCNB2, MAD2L1, PLK1, SPAG5, NCAPG2, CDCA5, HELLS, ERCC6L
**278**	3.68E-22	mitotic cell cycle	CDK1, CCNF, NUF2, TPX2, CDC20, BIRC5, AURKA, ANLN, CEP55, UBE2C, CCNB1, SPC25, CCNB2, MAD2L1, PLK1, SPAG5, NCAPG2, CDCA5, HELLS, ERCC6L
**48522**	2.20E-10	positive regulation of cellular process	BID, STEAP3, EGR1, CDK1, HMGB2, HSP90AA1, SOCS3, LGALS1, SNCA, TPX2, CDC20, BIRC5, ZBTB16, PMAIP1, ALDH3A1, RAD51, CCNB1, LCN2, FOS, RRM2, JUN, FCER1G, HSPD1, MYC, TOP2A
**32502**	2.77E-10	developmental process	XDH, STIL, HMGB2, ZBTB16, TTN, FOS, SHISA2, NCAPG2, KRT8, TOP2A, MYC, HELLS, TRIP13, EGR1, CDK1, HSP90AA1, LGALS3, SOCS3, LGALS1, CCNF, TPX2, CDC20, BIRC5, RACGAP1, TACC3, ECT2, RAD51, CCNB1, UHRF1, CCNB2, JUN, GADD45G
**48518**	4.06E-10	positive regulation of biological process	STEAP3, BID, HMGB2, SNCA, PMAIP1, HSPA1B, ZBTB16, ALDH3A1, FOS, FCER1G, TOP2A, MYC, EGR1, CDK1, HSP90AA1, SOCS3, LGALS1, TPX2, BIRC5, CDC20, RAD51, CCNB1, LCN2, JUN, RRM2, HSPD1
**65007**	4.06E-10	biological regulation	STEAP3, STIL, SNCA, S100A9, PMAIP1, FOS, CDKN2C, LTF, MYC, TOP2A, EGR1, CDK1, HSP90AA1, SOCS3, TPX2, UBE2C, TACC3, ECT2, MCM5, RAD51, CTSK, UHRF1, MAD2L1, KRT18, JUN, RRM2, GADD45G, BID, XDH, HMGB2, HSPA1B, ZBTB16, ALDH3A1, APLNR, KRT8, CLEC2D, FCER1G, ASF1B, HSPA8, HELLS, TNXB, LGALS1, NUF2, BIRC5, CDC20, RACGAP1, LCN2, CCNB1, CCNB2, HSPD1
**7275**	1.25E-09	multicellular organismal development	XDH, STIL, HMGB2, ZBTB16, TTN, FOS, SHISA2, NCAPG2, KRT8, TOP2A, MYC, HELLS, EGR1, CDK1, HSP90AA1, LGALS3, SOCS3, LGALS1, CCNF, TPX2, CDC20, BIRC5, RACGAP1, TACC3, RAD51, CCNB1, UHRF1, CCNB2, JUN, GADD45G

Table continues to the next page.

GO-ID	corrected p-value	Description	Genes in test set
**48856**	2.27E-09	anatomical structure development	XDH, STIL, HMGB2, ZBTB16, TTN, FOS, NCAPG2, KRT8, MYC, HELLS, TRIP13, EGR1, CDK1, HSP90AA1, LGALS3, SOCS3, LGALS1, CCNF, TPX2, CDC20, RACGAP1, TACC3, ECT2, RAD51, CCNB1, CCNB2, JUN, GADD45G
**50794**	9.76E-09	regulation of cellular process	XDH, BID, STEAP3, STIL, HMGB2, S100A9, SNCA, ZBTB16, HSPA1B, PMAIP1, ALDH3A1, APLNR, FOS, CDKN2C, KRT8, CLEC2D, FCER1G, ASF1B, TOP2A, MYC, HELLS, HSPA8, EGR1, CDK1, TNXB, HSP90AA1, SOCS3, LGALS1, TPX2, CDC20, BIRC5, RACGAP1, TACC3, ECT2, MCM5, RAD51, CCNB1, LCN2, UHRF1, KRT18, MAD2L1, JUN, RRM2, GADD45G, HSPD1
**50896**	1.01E-08	response to stimulus	XDH, HMGB2, S100A8, S100A9, SNCA, PMAIP1, HSPA1B, ALDH3A1, FOS, HSPH1, KRT8, CLEC2D, FCER1G, MYC, HSPA8, TRIP13, HSPA9, EGR1, CDK1, HSP90AA1, TACC3, RAD51, LCN2, UHRF1, JUN, GADD45G, HSPB1, HSPD1
**50789**	1.20E-08	regulation of biological process	XDH, BID, STEAP3, STIL, HMGB2, S100A9, SNCA, ZBTB16, HSPA1B, PMAIP1, ALDH3A1, APLNR, FOS, CDKN2C, KRT8, CLEC2D, FCER1G, ASF1B, TOP2A, MYC, HELLS, HSPA8, EGR1, CDK1, TNXB, HSP90AA1, SOCS3, LGALS1, TPX2, CDC20, BIRC5, RACGAP1, TACC3, UBE2C, ECT2, MCM5, RAD51, CCNB1, LCN2, UHRF1, KRT18, MAD2L1, JUN, RRM2, GADD45G, HSPD1
**6950**	1.31E-08	response to stress	CDK1, HSP90AA1, SNCA, PMAIP1, HSPA1B, TACC3, RAD51, ALDH3A1, LCN2, HSPH1, UHRF1, JUN, KRT8, CLEC2D, FCER1G, HSPB1, HSPD1, HSPA8, TRIP13, HSPA9
**42981**	1.91E-07	regulation of apoptosis	STEAP3, BID, STIL, SNCA, BIRC5, PMAIP1, HSPA1B, ZBTB16, RAD51, JUN, FCER1G, HSPD1, MYC, HELLS
**43067**	2.19E-07	regulation of programmed cell death	STEAP3, BID, STIL, SNCA, BIRC5, PMAIP1, HSPA1B, ZBTB16, RAD51, JUN, FCER1G, HSPD1, MYC, HELLS
**10941**	2.71E-07	regulation of cell death	STEAP3, BID, STIL, SNCA, BIRC5, PMAIP1, HSPA1B, ZBTB16, RAD51, JUN, FCER1G, HSPD1, MYC, HELLS
**22607**	2.76E-07	cellular component assembly	CDK1, TNXB, TPX2, TTN, CCNB1, LCN2, CENPA, RRM2, KRT8, TUBA4A, ASF1B, MYC, HELLS
**32501**	3.53E-07	multicellular organismal process	XDH, STIL, HMGB2, SNCA, ZBTB16, TTN, FOS, APLNR, SHISA2, NCAPG2, KRT8, TOP2A, MYC, HELLS, TRIP13, EGR1, CDK1, TNXB, HSP90AA1, LGALS3, SOCS3, LGALS1, CCNF, TPX2, CDC20, BIRC5, RACGAP1, TACC3, RAD51, CCNB1, UHRF1, CTSK, CCNB2, JUN, GADD45G
**42221**	4.31E-07	response to chemical stimulus	EGR1, XDH, CDK1, HMGB2, HSP90AA1, S100A8, SNCA, S100A9, HSPA1B, ALDH3A1, LCN2, FOS, JUN, KRT8, FCER1G, HSPD1, MYC
**51716**	4.33E-07	cellular response to stimulus	EGR1, CDK1, SNCA, S100A9, PMAIP1, HSPA1B, RAD51, LCN2, FOS, UHRF1, JUN, FCER1G, MYC, TRIP13
**48523**	7.58E-07	negative regulation of cellular process	EGR1, STIL, SOCS3, LGALS1, SNCA, BIRC5, PMAIP1, HSPA1B, ZBTB16, RAD51, CCNB1, MAD2L1, CDKN2C, JUN, CLEC2D, FCER1G, HSPD1, MYC, HELLS
**48731**	8.69E-07	system development	XDH, EGR1, STIL, CDK1, HMGB2, HSP90AA1, LGALS3, SOCS3, LGALS1, CCNF, CDC20, ZBTB16, RACGAP1, TTN, TACC3, RAD51, FOS, CCNB2, JUN, KRT8, GADD45G, MYC, HELLS
**48519**	8.79E-07	negative regulation of biological process	EGR1, STIL, SOCS3, LGALS1, SNCA, BIRC5, ZBTB16, PMAIP1, HSPA1B, TACC3, RAD51, CCNB1, MAD2L1, CDKN2C, JUN, CLEC2D, FCER1G, HSPD1, MYC, HELLS
**51726**	1.05E-06	regulation of cell cycle	CDK1, MAD2L1, JUN, GADD45G, TPX2, BIRC5, TACC3, MYC, HSPA8
**44085**	1.48E-06	cellular component biogenesis	CDK1, TNXB, TPX2, TTN, CCNB1, LCN2, CENPA, RRM2, KRT8, TUBA4A, ASF1B, MYC, HELLS
**6323**	1.81E-06	DNA packaging	CDK1, CENPA, NCAPG2, ASF1B, TOP2A, HELLS
**48869**	2.42E-06	cellular developmental process	EGR1, HMGB2, HSP90AA1, LGALS3, SOCS3, LGALS1, TPX2, CDC20, ZBTB16, RACGAP1, TTN, TACC3, ECT2, RAD51, CCNB1, JUN, KRT8, GADD45G, HELLS, TRIP13
**9790**	2.77E-06	embryonic development	CCNB1, STIL, CCNB2, NCAPG2, SOCS3, KRT8, BIRC5, ZBTB16, RACGAP1, TTN, TOP2A, MYC, RAD51
**71103**	3.74E-06	DNA conformation change	CDK1, CENPA, NCAPG2, ASF1B, TOP2A, HELLS
**6259**	5.03E-06	DNA metabolic process	UHRF1, RRM2, HSPA1B, TOP2A, MYC, HELLS, MCM5, TRIP13, TK1, RAD51
**43066**	6.41E-06	negative regulation of apoptosis	STIL, SNCA, FCER1G, BIRC5, HSPD1, HSPA1B, MYC, HELLS, RAD51
**1836**	6.93E-06	release of cytochrome c from mitochondria	BID, JUN, PMAIP1, MYC
**43069**	6.93E-06	negative regulation of programmed cell death	STIL, SNCA, FCER1G, BIRC5, HSPD1, HSPA1B, MYC, HELLS, RAD51

Table continues to the next page.

GO-ID	corrected p-value	Description	Genes in test set
**30154**	6.93E-06	cell differentiation	EGR1, HMGB2, HSP90AA1, LGALS3, SOCS3, LGALS1, TPX2, CDC20, ZBTB16, RACGAP1, TTN, TACC3, RAD51, CCNB1, JUN, GADD45G, KRT8, HELLS, TRIP13
**51789**	7.98E-06	response to protein stimulus	EGR1, FOS, HSP90AA1, HSPD1, HSPA1B, MYC
**60548**	8.60E-06	negative regulation of cell death	STIL, SNCA, FCER1G, BIRC5, HSPD1, HSPA1B, MYC, HELLS, RAD51
**51239**	9.55E-06	regulation of multicellular organismal process	EGR1, XDH, CDK1, HSP90AA1, LGALS1, SNCA, CDC20, HSPA1B, RAD51, CCNB1, JUN, CLEC2D, FCER1G, HSPD1, MYC
**6915**	2.63E-05	apoptosis	BID, STEAP3, KRT18, JUN, KRT8, GADD45G, TPX2, BIRC5, PMAIP1, MYC
**8637**	2.66E-05	apoptotic mitochondrial changes	BID, JUN, PMAIP1, MYC
**6457**	2.79E-05	protein folding	HSPH1, HSP90AA1, FKBP5, HSPD1, HSPA8, HSPA9
**12501**	2.88E-05	programmed cell death	BID, STEAP3, KRT18, JUN, KRT8, GADD45G, TPX2, BIRC5, PMAIP1, MYC
**1701**	3.76E-05	in utero embryonic development	CCNB1, STIL, CCNB2, NCAPG2, SOCS3, KRT8, TTN, RAD51
**65003**	3.76E-05	macromolecular complex assembly	CCNB1, LCN2, CDK1, CENPA, RRM2, TUBA4A, ASF1B, MYC, HELLS
**44260**	4.68E-05	cellular macromolecule metabolic process	FKBP5, AURKA, HSPA1B, TK1, FOS, HSPH1, ASF1B, TOP2A, MYC, HSPA8, HELLS, TRIP13, HSPA9, EGR1, CDK1, HSP90AA1, PBK, UBE2C, MCM5, RAD51, CCNB1, UHRF1, PLK1, JUN, RRM2, GADD45G, HSPD1, MELK
**43170**	4.92E-05	macromolecule metabolic process	FKBP5, AURKA, HSPA1B, TK1, FOS, HSPH1, ASF1B, TOP2A, MYC, HSPA8, HELLS, TRIP13, HSPA9, EGR1, CDK1, TNXB, HSP90AA1, PBK, UBE2C, MCM5, RAD51, CCNB1, UHRF1, CTSK, PLK1, JUN, RRM2, GADD45G, HSPD1, MELK
**8219**	5.83E-05	cell death	BID, STEAP3, KRT18, JUN, KRT8, GADD45G, TPX2, BIRC5, PMAIP1, MYC
**43933**	6.34E-05	macromolecular complex subunit organization	CCNB1, LCN2, CDK1, CENPA, RRM2, TUBA4A, ASF1B, MYC, HELLS
**16265**	6.77E-05	death	BID, STEAP3, KRT18, JUN, KRT8, GADD45G, TPX2, BIRC5, PMAIP1, MYC
**33554**	8.99E-05	cellular response to stress	LCN2, CDK1, UHRF1, JUN, SNCA, HSPA1B, PMAIP1, TRIP13, RAD51
**51128**	9.70E-05	regulation of cellular component organization	CCNB1, LCN2, MAD2L1, HSP90AA1, LGALS1, S100A9, TPX2, FCER1G, CDC20
**51085**	1.30E-04	chaperone mediated protein folding requiring cofactor	HSPH1, HSPD1, HSPA8
**7005**	1.55E-04	mitochondrion organization	BID, JUN, SNCA, PMAIP1, MYC
**61077**	1.60E-04	chaperone-mediated protein folding	HSPH1, HSPD1, HSPA8
**7017**	1.65E-04	microtubule-based process	NUF2, TPX2, TUBA4A, BIRC5, TACC3, KIF20A
**51276**	1.67E-04	chromosome organization	CDK1, MAD2L1, CENPA, NCAPG2, HSPA1B, ASF1B, TOP2A, HELLS
**7126**	1.67E-04	meiosis	MKI67, PLK1, CKS2, TRIP13, RAD51
**51327**	1.67E-04	M phase of meiotic cell cycle	MKI67, PLK1, CKS2, TRIP13, RAD51
**51321**	1.73E-04	meiotic cell cycle	MKI67, PLK1, CKS2, TRIP13, RAD51
**6458**	1.79E-04	'de novo' protein folding	HSPH1, HSPD1, HSPA8
**51084**	1.79E-04	'de novo' posttranslational protein folding	HSPH1, HSPD1, HSPA8
**6916**	1.83E-04	anti-apoptosis	BIRC5, HSPD1, HSPA1B, MYC, HELLS
**7059**	2.26E-04	chromosome segregation	MAD2L1, NUF2, BIRC5, TOP2A
**7281**	2.44E-04	germ cell development	CCNB1, HMGB2, TPX2, ZBTB16, TRIP13

Table continues to the next page.

GO-ID	corrected p-value	Description	Genes in test set
**50793**	2.44E-04	regulation of developmental process	CCNB1, XDH, CDK1, HSP90AA1, SOCS3, LGALS1, JUN, CLEC2D, CDC20, MYC, RAD51
**30261**	2.49E-04	chromosome condensation	CDK1, NCAPG2, TOP2A
**44237**	2.57E-04	cellular metabolic process	XDH, FKBP5, SNCA, AURKA, HSPA1B, ALDH3A1, TK1, FOS, HSPH1, ASF1B, TOP2A, MYC, HELLS, HSPA8, TRIP13, HSPA9, EGR1, CDK1, TNXB, HSP90AA1, PBK, UBE2C, MCM5, RAD51, CCNB1, UHRF1, PLK1, JUN, RRM2, GADD45G, HSPD1, MELK
**43523**	2.80E-04	regulation of neuron apoptosis	JUN, SNCA, BIRC5, PMAIP1, RAD51
**910**	2.87E-04	cytokinesis	PRC1, PLK1, ANLN
**19222**	2.94E-04	regulation of metabolic process	EGR1, CDK1, HMGB2, TNXB, HSP90AA1, SOCS3, SNCA, S100A9, TPX2, ZBTB16, UBE2C, MCM5, CCNB1, LCN2, FOS, UHRF1, JUN, GADD45G, ASF1B, MYC, TOP2A, HELLS
**42127**	3.20E-04	regulation of cell proliferation	CCNB1, CDK1, CDKN2C, RRM2, JUN, CDC20, ZBTB16, PMAIP1, MYC, ALDH3A1
**10033**	3.25E-04	response to organic substance	EGR1, CDK1, FOS, HMGB2, HSP90AA1, SNCA, HSPD1, HSPA1B, MYC, ALDH3A1
**42493**	3.25E-04	response to drug	LCN2, FOS, JUN, SNCA, ALDH3A1
**7010**	3.34E-04	cytoskeleton organization	KRT8, S100A9, NUF2, TPX2, BIRC5, TACC3, TTN
**60255**	3.40E-04	regulation of macromolecule metabolic process	EGR1, CDK1, HMGB2, TNXB, SOCS3, SNCA, S100A9, TPX2, ZBTB16, UBE2C, MCM5, CCNB1, LCN2, FOS, UHRF1, JUN, ASF1B, MYC, TOP2A, HELLS
**7006**	3.66E-04	mitochondrial membrane organization	BID, SNCA, PMAIP1
**44238**	5.16E-04	primary metabolic process	XDH, FKBP5, SNCA, AURKA, HSPA1B, TK1, FOS, HSPH1, ASF1B, TOP2A, MYC, HELLS, HSPA8, TRIP13, HSPA9, EGR1, CDK1, TNXB, HSP90AA1, PBK, UBE2C, MCM5, RAD51, CCNB1, UHRF1, CTSK, PLK1, JUN, RRM2, GADD45G, HSPD1, MELK
**43009**	6.02E-04	chordate embryonic development	CCNB1, STIL, CCNB2, NCAPG2, SOCS3, KRT8, TTN, RAD51
**8152**	6.02E-04	metabolic process	STEAP3, XDH, FKBP5, SNCA, AURKA, HSPA1B, TK1, ALDH3A1, FOS, HSPH1, LTF, ASF1B, TOP2A, MYC, HELLS, HSPA8, TRIP13, HSPA9, EGR1, CDK1, TNXB, HSP90AA1, PBK, UBE2C, MCM5, RAD51, CCNB1, UHRF1, CTSK, PLK1, JUN, RRM2, GADD45G, HSPD1, MELK
**9792**	6.38E-04	embryonic development ending in birth or egg hatching	CCNB1, STIL, CCNB2, NCAPG2, SOCS3, KRT8, TTN, RAD51
**9893**	7.81E-04	positive regulation of metabolic process	CCNB1, LCN2, EGR1, CDK1, FOS, HMGB2, HSP90AA1, JUN, TPX2, TOP2A, MYC
**45321**	8.03E-04	leukocyte activation	EGR1, GADD45G, SNCA, FCER1G, HSPD1, HELLS
**51094**	1.01E-03	positive regulation of developmental process	CCNB1, CDK1, HSP90AA1, SOCS3, JUN, MYC, RAD51
**48513**	1.04E-03	organ development	EGR1, XDH, STIL, CDK1, HMGB2, SOCS3, CCNF, TTN, TACC3, CCNB2, JUN, KRT8, GADD45G, MYC, HELLS
**8283**	1.22E-03	cell proliferation	UHRF1, MKI67, NCAPG2, RACGAP1, TACC3, HELLS
**2682**	1.29E-03	regulation of immune system process	HSP90AA1, JUN, SNCA, CLEC2D, FCER1G, HSPD1, HSPA1B
**70887**	1.30E-03	cellular response to chemical stimulus	LCN2, EGR1, S100A9, SNCA, FCER1G, MYC
**1775**	1.32E-03	cell activation	EGR1, GADD45G, SNCA, FCER1G, HSPD1, HELLS
**226**	1.35E-03	microtubule cytoskeleton organization	NUF2, TPX2, BIRC5, TACC3
**51240**	1.37E-03	positive regulation of multicellular organismal process	CCNB1, CDK1, SNCA, FCER1G, HSPD1, MYC
**10604**	1.44E-03	positive regulation of macromolecule metabolic process	CCNB1, LCN2, EGR1, CDK1, FOS, HMGB2, JUN, TPX2, TOP2A, MYC
**51173**	1.49E-03	positive regulation of nitrogen compound metabolic process	CCNB1, EGR1, CDK1, FOS, HMGB2, HSP90AA1, JUN, TOP2A, MYC

Table continues to the next page.

GO-ID	corrected p-value	Description	Genes in test set
**7346**	1.49E-03	regulation of mitotic cell cycle	CDK1, MAD2L1, TPX2, MYC
**8284**	1.49E-03	positive regulation of cell proliferation	CCNB1, CDK1, RRM2, JUN, CDC20, MYC, ALDH3A1
**1933**	1.53E-03	negative regulation of protein amino acid phosphorylation	CCNB1, JUN, SNCA
**9628**	1.53E-03	response to abiotic stimulus	KRT8, HSPB1, HSPA1B, PMAIP1, MYC, RAD51
**1932**	1.72E-03	regulation of protein amino acid phosphorylation	CCNB1, TNXB, SOCS3, JUN, SNCA
**31325**	1.72E-03	positive regulation of cellular metabolic process	CCNB1, EGR1, CDK1, FOS, HMGB2, HSP90AA1, JUN, TPX2, TOP2A, MYC
**43065**	1.92E-03	positive regulation of apoptosis	BID, STEAP3, JUN, ZBTB16, PMAIP1, MYC
**45595**	1.98E-03	regulation of cell differentiation	XDH, HSP90AA1, SOCS3, LGALS1, JUN, CLEC2D, CDC20, RAD51
**43068**	1.99E-03	positive regulation of programmed cell death	BID, STEAP3, JUN, ZBTB16, PMAIP1, MYC
**7127**	1.99E-03	meiosis I	CKS2, TRIP13, RAD51
**2376**	1.99E-03	immune system process	EGR1, CCNB2, GADD45G, S100A9, SNCA, FCER1G, HSPD1, TACC3, HELLS
**6974**	2.05E-03	response to DNA damage stimulus	CDK1, UHRF1, HSPA1B, PMAIP1, TRIP13, RAD51
**31328**	2.06E-03	positive regulation of cellular biosynthetic process	EGR1, CDK1, FOS, HMGB2, HSP90AA1, JUN, TPX2, TOP2A, MYC
**48610**	2.11E-03	reproductive cellular process	CCNB1, HMGB2, TPX2, ZBTB16, TRIP13
**10942**	2.11E-03	positive regulation of cell death	BID, STEAP3, JUN, ZBTB16, PMAIP1, MYC
**9891**	2.25E-03	positive regulation of biosynthetic process	EGR1, CDK1, FOS, HMGB2, HSP90AA1, JUN, TPX2, TOP2A, MYC
**51246**	2.38E-03	regulation of protein metabolic process	CCNB1, TNXB, SOCS3, JUN, SNCA, TPX2, UBE2C
**31323**	2.44E-03	regulation of cellular metabolic process	EGR1, CDK1, HMGB2, TNXB, HSP90AA1, SOCS3, SNCA, TPX2, ZBTB16, MCM5, CCNB1, FOS, UHRF1, JUN, GADD45G, ASF1B, MYC, TOP2A, HELLS
**48477**	2.50E-03	oogenesis	CCNB1, TPX2, TRIP13
**32504**	2.54E-03	multicellular organism reproduction	CCNB1, XDH, HMGB2, TPX2, ZBTB16, RACGAP1, TRIP13
**48609**	2.54E-03	reproductive process in a multicellular organism	CCNB1, XDH, HMGB2, TPX2, ZBTB16, RACGAP1, TRIP13
**6461**	2.54E-03	protein complex assembly	CCNB1, LCN2, CDK1, RRM2, TUBA4A, MYC
**70271**	2.54E-03	protein complex biogenesis	CCNB1, LCN2, CDK1, RRM2, TUBA4A, MYC
**51054**	2.57E-03	positive regulation of DNA metabolic process	CDK1, HMGB2, JUN
**44267**	2.66E-03	cellular protein metabolic process	CDK1, HSP90AA1, FKBP5, AURKA, PBK, UBE2C, CCNB1, HSPH1, PLK1, GADD45G, HSPD1, MELK, HSPA8, HSPA9
**7131**	2.81E-03	reciprocal meiotic recombination	TRIP13, RAD51
**60045**	2.81E-03	positive regulation of cardiac muscle cell proliferation	CCNB1, CDK1
**19538**	3.18E-03	protein metabolic process	CDK1, HSP90AA1, FKBP5, AURKA, PBK, UBE2C, CCNB1, HSPH1, CTSK, PLK1, GADD45G, HSPD1, MYC, MELK, HSPA8, HSPA9

Table continues to the next page.

GO-ID	corrected p-value	Description	Genes in test set
**9607**	3.18E-03	response to biotic stimulus	LCN2, HSP90AA1, KRT8, FCER1G, HSPD1, HSPA1B
**31497**	3.39E-03	chromatin assembly	CENPA, ASF1B, HELLS
**65008**	3.39E-03	regulation of biological quality	XDH, BID, EGR1, APLNR, CTSK, CCNB2, JUN, SNCA, NUF2, LTF, HSPA1B, TACC3
**16310**	3.51E-03	phosphorylation	CCNB1, CDK1, PLK1, GADD45G, SNCA, AURKA, PBK, MELK, TK1
**80090**	3.61E-03	regulation of primary metabolic process	EGR1, CDK1, HMGB2, TNXB, SOCS3, SNCA, TPX2, ZBTB16, UBE2C, MCM5, CCNB1, FOS, UHRF1, JUN, ASF1B, MYC, TOP2A, HELLS
**31399**	3.76E-03	regulation of protein modification process	CCNB1, TNXB, SOCS3, JUN, SNCA
**65004**	3.92E-03	protein-DNA complex assembly	CENPA, ASF1B, HELLS
**10628**	3.92E-03	positive regulation of gene expression	LCN2, EGR1, CDK1, FOS, HMGB2, JUN, TOP2A, MYC
**51254**	3.92E-03	positive regulation of RNA metabolic process	CCNB1, EGR1, FOS, HMGB2, JUN, TOP2A, MYC
**33209**	3.94E-03	tumor necrosis factor-mediated signaling pathway	KRT18, KRT8
**42326**	4.04E-03	negative regulation of phosphorylation	CCNB1, JUN, SNCA
**7276**	4.04E-03	gamete generation	CCNB1, HMGB2, TPX2, ZBTB16, RACGAP1, TRIP13
**9605**	4.18E-03	response to external stimulus	FOS, S100A8, JUN, S100A9, FCER1G, MYC
**31400**	4.18E-03	negative regulation of protein modification process	CCNB1, JUN, SNCA
**45935**	4.47E-03	positive regulation of nucleobase, nucleoside, nucleotide and nucleic acid metabolic process	CCNB1, EGR1, CDK1, FOS, HMGB2, JUN, TOP2A, MYC
**45936**	4.57E-03	negative regulation of phosphate metabolic process	CCNB1, JUN, SNCA
**10563**	4.57E-03	negative regulation of phosphorus metabolic process	CCNB1, JUN, SNCA
**32268**	5.00E-03	regulation of cellular protein metabolic process	CCNB1, TNXB, SOCS3, JUN, SNCA, TPX2
**60706**	5.19E-03	cell differentiation involved in embryonic placenta development	SOCS3, KRT8
**45214**	5.19E-03	sarcomere organization	KRT8, TTN
**43524**	5.19E-03	negative regulation of neuron apoptosis	SNCA, BIRC5, RAD51
**10557**	5.37E-03	positive regulation of macromolecule biosynthetic process	EGR1, CDK1, FOS, HMGB2, JUN, TPX2, TOP2A, MYC
**42325**	5.73E-03	regulation of phosphorylation	CCNB1, TNXB, SOCS3, JUN, GADD45G, SNCA
**55021**	5.73E-03	regulation of cardiac muscle tissue growth	CCNB1, CDK1
**55024**	5.73E-03	regulation of cardiac muscle tissue development	CCNB1, CDK1
**10165**	5.73E-03	response to X-ray	PMAIP1, RAD51
**60043**	5.73E-03	regulation of cardiac muscle cell proliferation	CCNB1, CDK1
**1556**	5.73E-03	oocyte maturation	CCNB1, TRIP13
**9889**	5.85E-03	regulation of biosynthetic process	EGR1, CDK1, HMGB2, HSP90AA1, SNCA, S100A9, TPX2, ZBTB16, MCM5, FOS, UHRF1, JUN, ASF1B, MYC, TOP2A, HELLS
**7292**	5.96E-03	female gamete generation	CCNB1, TPX2, TRIP13

Table continues to the next page.

GO-ID	corrected p-value	Description	Genes in test set
**7143**	6.48E-03	female meiosis	PLK1, TRIP13
**31344**	6.64E-03	regulation of cell projection organization	LCN2, HSP90AA1, LGALS1, CDC20
**51174**	6.74E-03	regulation of phosphorus metabolic process	CCNB1, TNXB, SOCS3, JUN, GADD45G, SNCA
**19220**	6.74E-03	regulation of phosphate metabolic process	CCNB1, TNXB, SOCS3, JUN, GADD45G, SNCA
**6366**	6.93E-03	transcription from RNA polymerase II promoter	FOS, JUN, MYC
**19953**	7.02E-03	sexual reproduction	CCNB1, HMGB2, TPX2, ZBTB16, RACGAP1, TRIP13
**60420**	7.08E-03	regulation of heart growth	CCNB1, CDK1
**45453**	7.08E-03	bone resorption	XDH, CTSK
**7283**	7.28E-03	spermatogenesis	HMGB2, TPX2, ZBTB16, RACGAP1, TRIP13
**48232**	7.28E-03	male gamete generation	HMGB2, TPX2, ZBTB16, RACGAP1, TRIP13
**34622**	7.66E-03	cellular macromolecular complex assembly	CENPA, TUBA4A, ASF1B, HELLS
**6333**	8.56E-03	chromatin assembly or disassembly	CENPA, ASF1B, HELLS
**7051**	8.73E-03	spindle organization	TPX2, TACC3
**2706**	8.75E-03	regulation of lymphocyte mediated immunity	CLEC2D, FCER1G, HSPA1B
**51052**	8.75E-03	regulation of DNA metabolic process	CDK1, HMGB2, JUN
**3006**	8.82E-03	reproductive developmental process	CCNB1, HMGB2, TPX2, ZBTB16, TRIP13
**45944**	8.82E-03	positive regulation of transcription from RNA polymerase II promoter	EGR1, FOS, HMGB2, JUN, TOP2A, MYC
**7093**	9.47E-03	mitotic cell cycle checkpoint	CDK1, MAD2L1
**8104**	9.51E-03	protein localization	BID, STEAP3, NUF2, ZBTB16, PMAIP1, TACC3, KIF20A, HSPA9
